# Supplementary material for: Resistin Promotes Nasopharyngeal Carcinoma Metastasis through TLR4-Mediated Activation of p38 MAPK/NF-κB Signaling Pathway
Source: Cancers (Basel). 2022 Dec 5;14(23):6003. doi: 10.3390/cancers14236003 (PMC9737889; doi:10.3390/cancers14236003)
Supplement: Supplementary file 1 [file cancers-14-06003-s001.zip › cancers-2026000-supplementary.pdf]

## Supplementary Methods

### Bioinformatics analyses of TLR4 expression

Messenger RNA (mRNA) expression data for 566 head and neck squamous cell carcinoma (HNSC) samples were downloaded from The Cancer Genome Atlas (TCGA) data portal (https://xenabrowser.net/datapages/). According to the anatomic neoplasm subdivision, this included 44 tonsil, 9 oropharynx, 143 oral tongue, 87 oral cavity, 3 lip, 128 larynx, 10 hypopharynx, 7 hard palate, 66 floor of mouth, 22 buccal mucosa, 29 base of tongue and 18 alveolar ridge tumors.

The microarray gene expression profiling data included GSE12452 (10 normal controls and 31 NPC samples) [1], GSE53819 (21 normal controls and 18 NPC samples) [2], GSE61218 (21 normal controls and 18 NPC samples) [3], GSE64634 (4 normal controls and 12 NPC samples) [4], GSE103611 (48 NPC samples) [5], GSE132112 (95 NPC samples) [6] and GSE13597 (3 normal controls and 25 NPC samples) [7]. The RNA-seq data of NPC samples included GSE102349 (113 NPC samples) [8] and GSE68799 (4 normal controls and 42 NPC samples). These data were downloaded from the Gene Expression Omnibus (GEO) database.

### References

1. Sengupta, S.; den Boon, J.A.; Chen, I.H.; Newton, M.A.; Dahl, D.B.; Chen, M.; Cheng, Y.-J.; Westra, W.H.; Chen, C.-J.; Hildesheim, A.; et al. Genome-Wide Expression Profiling Reveals EBV-Associated Inhibition of MHC Class I Expression in Nasopharyngeal Carcinoma. *Cancer Res.* **2006**, *66*, 7999.
2. Bao, Y.-N.; Cao, X.; Luo, D.-H.; Sun, R.; Peng, L.-X.; Wang, L.; Yan, Y.-P.; Zheng, L.-S.; Xie, P.; Cao, Y.; et al. Urokinase-type plasminogen activator receptor signaling is critical in nasopharyngeal carcinoma cell growth and metastasis. *Cell cycle* **2014**, *13*, 1958-69.
3. Fan, C.; Wang, J.; Tang, Y.; Zhang, S.; Xiong, F.; Guo, C.; Zhou, Y.; Li, Z.; Li, X.; Li, Y.; et al. Upregulation of long non-coding RNA LOC284454 may serve as a new serum diagnostic biomarker for head and neck cancers. *BMC Cancer* **2020**, *20*, 917.
4. Bo, H.; Gong, Z.; Zhang, W.; Li, X.; Zeng, Y.; Liao, Q.; Chen, P.; Shi, L.; Lian, Y.; Jing, Y.; et al. Upregulated long non-coding RNA AFAP1-AS1 expression is associated with progression

and poor prognosis of nasopharyngeal carcinoma. *Oncotarget* **2015**, *6*, 20404-18.
5. Tang, X.-R.; Li, Y.-Q.; Liang, S.-B.; Jiang, W.; Liu, F.; Ge, W.-X.; Tang, L.-L.; Mao, Y.-P.; He, Q.-M.; Yang, X.-J.; et al. Development and validation of a gene expression-based signature to predict distant metastasis in locoregionally advanced nasopharyngeal carcinoma: a retrospective, multicentre, cohort study. *Lancet Oncol.* **2018**, *19*, 382-393.
6. Lei, Y.; Li, Y.-Q.; Jiang, W.; Hong, X.-H.; Ge, W.-X.; Zhang, Y.; Hu, W.-H.; Wang, Y.-Q.; Liang, Y.-L.; Li, J.-Y.; et al. A Gene-Expression Predictor for Efficacy of Induction Chemotherapy in Locoregionally Advanced Nasopharyngeal Carcinoma. *J. Natl. Cancer Inst.* **2021**, *113*, 471-480.
7. Bose, S.; Yap, L.-F.; Fung, M.; Starzcynski, J.; Saleh, A.; Morgan, S.; Dawson, C.; Chukwuma, M.B.; Maina, E.; Buettner, M.; et al. The ATM tumour suppressor gene is down-regulated in EBV-associated nasopharyngeal carcinoma. *J. Pathol.* **2009**, *217*, 345-52.
8. Zhang, L.; MacIsaac, K.D.; Zhou, T.; Huang, P.-Y.; Xin, C.; Dobson, J.R.; Yu, K.; Chiang, D.Y.; Fan, Y.; Pelletier, M.; et al. Genomic Analysis of Nasopharyngeal Carcinoma Reveals TME-Based Subtypes. *Mol. Cancer Res.* **2017**, *15*, 1722.

**Supplementary Figure**

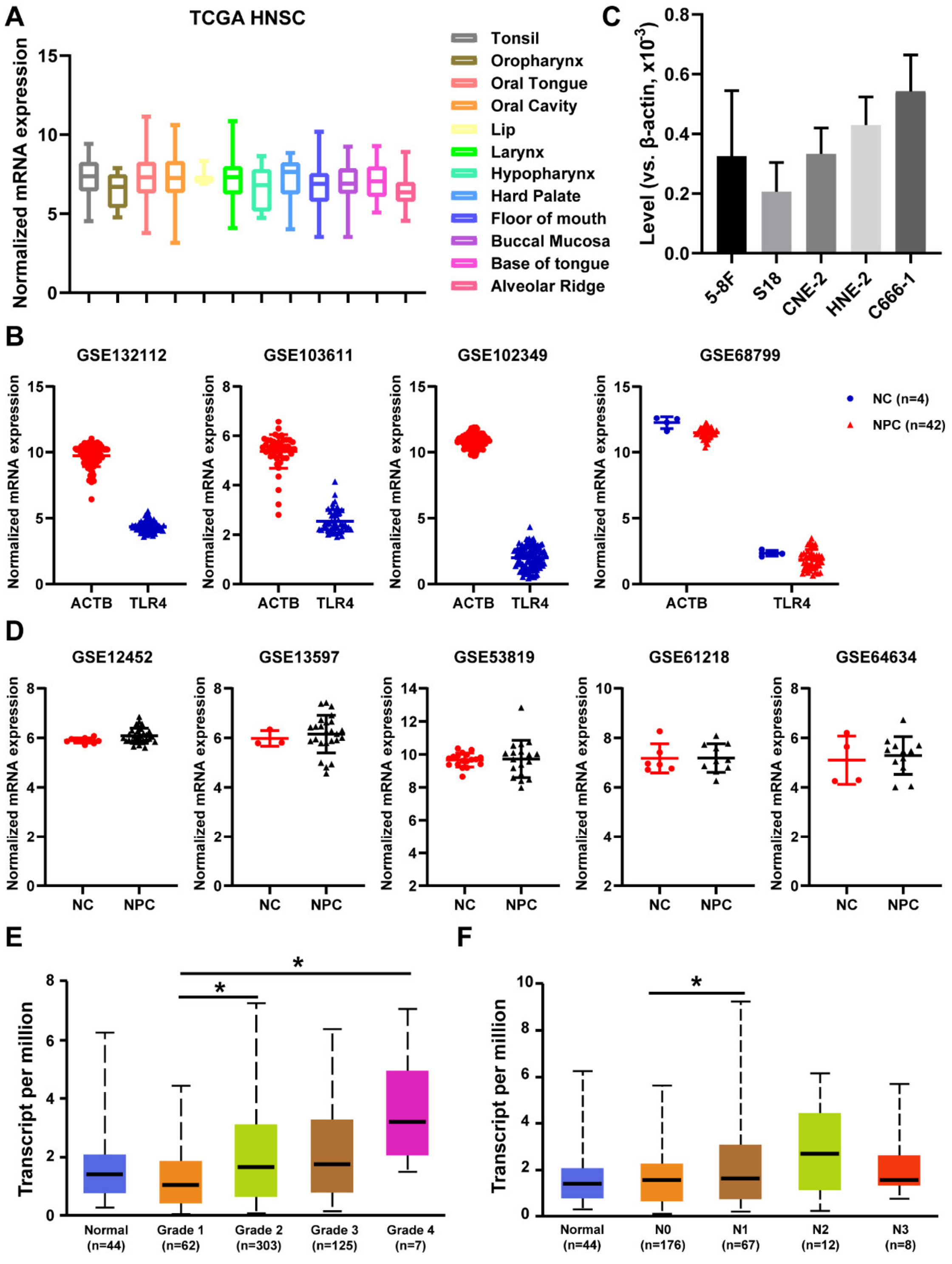


**Figure S1. The expression of TLR4 in human HNSC and NPC.**

**A** Box plots (derived from TCGA RNA-sequencing dataset) showing the expression of TLR4 in head and neck squamous cell carcinoma (HNSC). The boxes represent the 25th and 75th percentiles, the lines

represent the median, and whiskers show the minimum and maximum points. **B** The mRNA expression levels of TLR4 were analyzed in NPC tissues from the GEO datasets. **C** Expression of TLR4 was determined by qRT-PCR in NPC cell lines. **D** The relative mRNA expression of TLR4 in normal and NPC samples from GEO datasets. **E** Box plots showing the expression of TLR4 in normal and different HNSC grade samples. **F** Box plots showing the expression of TLR4 in normal and HNSC of lymph node metastasis samples. Data are expressed as normalized expression units. Data are presented as mean ± SD. *$P < 0.05$.

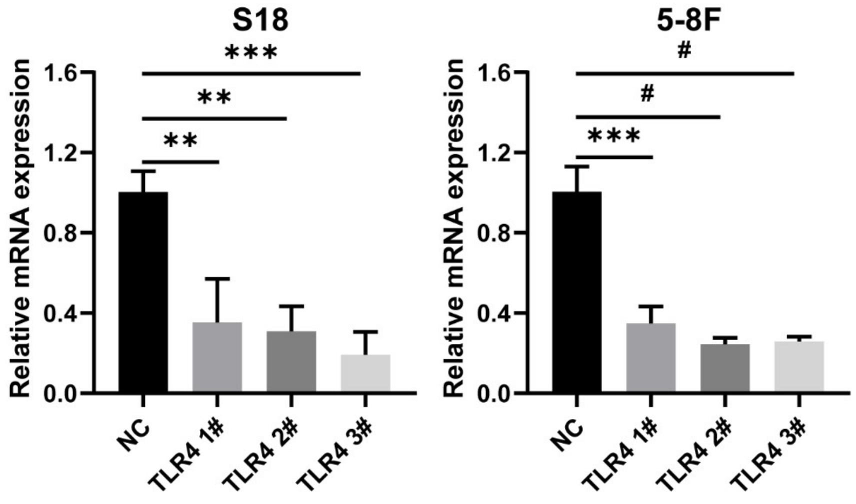


**Figure S2. Knockdown expression of TLR4 in NPC cells.**

S18 and 5-8F cells were transfected with 50 μM siRNAs of NC, the relative expression of each TLR4 mRNA against β-actin was measured with qRT-PCR. Data are presented as mean ± SD. **$P < 0.01$, ***$P < 0.001$, $^{\#}P < 0.0001$.

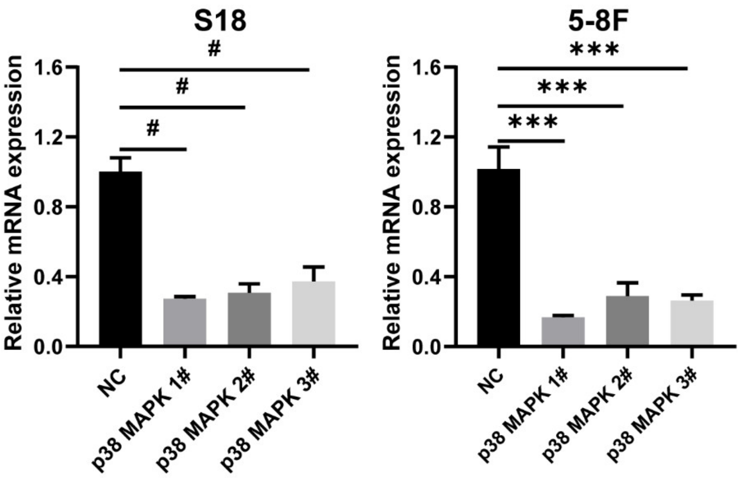


**Figure S3. Knockdown expression of p38 MAPK in NPC cells.**

S18 and 5-8F cells were transfected with 50 μM siRNAs of NC, TLR4. The relative expression of each p38 MAPK mRNA against β-actin was measured with qRT-PCR. Data are presented as mean ± SD. $^{***}P < 0.001$, $^{\#}P < 0.0001$.

**Supplementary Tables**

**Table S1. Sequences of small interfering RNA used in transfection.**

| Target gene | siRNA sequences |
|---|---|
| TLR4-siRNA#1 sense | 5'- GTGCAATTTGACCATTGAA -3' |
| TLR4-siRNA#2 sense | 5'- TGGTGAGTGTGACTATTGA -3' |
| TLR4-siRNA#3 sense | 5'- CTACTACCTCGATGATATT -3' |
| p38 MAPK-siRNA#1 sense | 5'- AGTCCATCATTCATGCGAA -3' |
| p38 MAPK-siRNA#2 sense | 5'- GCGGTTACTTAAACATATG -3' |
| p38 MAPK-siRNA#3 sense | 5'- CTCCGAGGTCTAAAGTATA -3' |

**Table S2. Sequences of primers used in quantitative RT-PCR.**

| Target gene | primer | primer sequence |
|---|---|---|
| ACTB | F | 5'- CCTGTACGCCAACACAGTGC -3' |
| | R | 5'- ATACTCCTGCTTGCTGATCC -3' |
| TLR4 | F | 5'- AGTTGATCTACCAAGCCTTGAGT -3' |
| | R | 5'- GCTGGTTGTCCCAAAATCACTTT -3' |
| p38 MAPK | F | 5'- TCAGTCCATCATTCATGCGAAA -3' |
| | R | 5'- AACGTCCAACAGACCAATCAC -3' |

**Table S3. Antibodies list.**

| Antibodies | Source | Identifier |
|---|---|---|
| Anti-β-Actin | Sigma | Cat#A2228 |
| Anti-α-Tubulin | Sigma | Cat#T6074 |
| Anti-Histone H3 | Cell Signaling Technology | Cat#4499 |
| Anti-E-cadherin | Cell Signaling Technology | Cat#3195 |
| Anti-N-cadherin | Cell Signaling Technology | Cat#13116 |
| Anti-MMP-2 | Abcam | Cat#ab92536 |
| Anti-MMP-9 | Abcam | Cat#ab58803 |
| Anti-ZEB1 | Cell Signaling Technology | Cat#3396 |
| Anti-Slug | Cell Signaling Technology | Cat#9585 |
| Anti-Snail | Thermo Fisher Scientific | Cat#MA5-14801 |
| Anti-Claudin-1 | Cell Signaling Technology | Cat#13255 |
| Anti-Vimentin | Cell Signaling Technology | Cat#5741 |
| Anti-ZO-1 | Cell Signaling Technology | Cat#8193 |
| Anti-β-catenin | Cell Signaling Technology | Cat#8480 |
| Anti-AKT | Cell Signaling Technology | Cat#9272 |
| Anti-p-AKT | Cell Signaling Technology | Cat#4060 |
| Anti-p38 | Cell Signaling Technology | Cat#8690 |
| Anti-p-p38 | Cell Signaling Technology | Cat#4511 |
| Anti-p65 | Cell Signaling Technology | Cat#8242 |
| Anti-p-p65 | Cell Signaling Technology | Cat#3033 |
| Anti-p50 | Cell Signaling Technology | Cat#13586 |
| Anti-Erk1/2 | Cell Signaling Technology | Cat#4695 |
| Anti-p-Erk1/2 | Cell Signaling Technology | Cat#4370 |

| | | |
|---|---|---|
| Anti-IκBα | Cell Signaling Technology | Cat#4814 |
| Anti-p-IκBα | Cell Signaling Technology | Cat#9246 |
| Goat anti-mouse-HRP | Jackson ImmunoResearch | Cat#115-035-003 |
| Goat anti-rabbit-HRP | Jackson ImmunoResearch | Cat#111-035-003 |
| Goat anti-Rabbit Alexa 555 | Cell Signaling Technology | Cat#4413 |
